# Prevalence and biomarker profiles of HBV/HDV coinfection and occult HBV infection among patients attending Bimbilla Hospital, Northern Ghana

**DOI:** 10.1186/s12879-026-13881-8

**Published:** 2026-06-25

**Authors:** Eric Nana Yaw Nyarko, Kenneth Tachi, Justice Kumi, Aliu Issahaku, Ebenezer Kwabena Amakye, Manfred Anim, Mildred Adusei-Poku, Nafisa Akua Assan, Isaac Kwame Sraku, Monica Adom, Isaac Kpabitey Lawer, Abass Abdul-Karim, Emmanuel Kwaku Ofori, Henry Asare-Anane, Christian Obirikorang

**Affiliations:** 1 Department of Chemical Pathology, University of Ghana Medical School, College of Health Sciences, University of Ghana, Accra, Ghana; 2 Department of Medicine and Therapeutics, University of Ghana Medical School, University of Ghana, Accra, Ghana; 3 Department of Clinical Pathology, Noguchi Memorial Institute for Medical Research, College of Health Sciences, University of Ghana, Accra, Ghana; 4 Department of Biomedical Laboratory Sciences, School of Allied Health Sciences, University for Development Studies, Tamale, Ghana; 5 Department of Gastroenterology, Hepatology, Infectious Diseases and Endocrinology, Hannover Medical School, Hannover, Germany; 6 Department of Medical Microbiology, University of Ghana Medical School, College of Health Sciences, University of Ghana, Accra, Ghana; 7 Tamale Zonal Public Health and Reference Laboratory, Tamale, Ghana; 8 Department of Molecular Medicine, School of Medicine and Dentistry, Kwame Nkrumah University of Science and Technology, Kumasi, Ghana; 9 Global Health and Infectious Diseases Group, Kumasi Centre for Collaborative Research, Kumasi, Ghana

**Keywords:** Occult hepatitis B infection (OBI), Hepatitis delta virus (HDV), Biomarkers, Bimbilla, Resource-limited settings

## Abstract

**Background & aims:**

Coinfection of hepatitis B virus (HBV) with hepatitis D virus (HDV) may exacerbate liver injury, particularly in resource-limited settings. In Ghana, data on HDV and OBI prevalence, as well as their hepatic impact, remain sparse. This study aimed to determine the prevalence of HDV and OBI using real-time PCR and evaluate their effects on liver-related biomarkers in patients at Bimbila Hospital, Northern Ghana.

**Methods:**

We conducted a cross-sectional study at Bimbilla Hospital, Northern Ghana, between May and December 2024, recruiting 60 consented patients with Hepatitis B Surface Antigen positive and 60 patients with HBsAg-negative status. HBV serology (HBsAg, HBeAg, anti-HBs, total anti-HBc) was performed using a 5-in-1 panel. HBV DNA and HDV RNA were detected by real-time PCR. HDV prevalence was determined among HBsAg-positive participants by real-time PCR detection of HDV RNA, while OBI prevalence was determined among HBsAg-negative participants by the presence of detectable HBV DNA using real-time PCR following serological screening. Liver biochemical, haematological, and inflammatory markers were measured, and fibrotic indices (APRI, FIB-4, PLR, GLR) were computed. Data were analysed using SPSS 26 and GraphPad Prism version 8.

**Results:**

HDV RNA was detected in 11/60 HBsAg-positive participants (18.3%, 95% CI 10.66–29.9). OBI occurred in 5/60 HBsAg-negative participants (8.3%, 95% CI 3.6–18.1). Compared to HBV-monoinfected individuals, HBV/HDV-coinfected patients had higher Gamma-Glutamyltransferase (GGT;45.0 vs. 37.0 IU/L, *p* < 0.05), lower Albumin/Globulin Ratio (A/G;1.21 vs. 1.34, *p* < 0.05), elevated Granulocyte-to-Lymphocyte Ratio (GLR; 2.60 vs. 1.87, *p* < 0.05), and higher Platelet-to-Lymphocyte Ratio (PLR; 143.00 vs. 89.87, *p* < 0.05). HBV/HDV coinfected patients also showed elevated Aspartate Aminotransferase (AST), Alanine Aminotransferase (ALT), total bilirubin (TBIL), IBL, AST/ALT ratio (de Ritis), and Aspartate Aminotransferase-to-Platelet Ratio Index (APRI) scores compared to those with OBI.

**Conclusion:**

In Northern Ghana, HDV coinfection may be associated with significant alterations in GGT, AST, ALT, bilirubin levels, de-Ritis ratio, APRI, PLR, and GLR, with concurrent reduction in the A/G ratio, suggesting significant hepatic and inflammatory disturbances, whereas OBI may show no comparable biomarker alterations.

**Clinical trial:**

Not applicable.

**Supplementary Information:**

The online version contains supplementary material available at https://doi.org/10.1186/s12879-026-13881-8.

## Introduction

Hepatitis B virus (HBV) infection remains a major global public health challenge, affecting over 296 million individuals and contributing significantly to liver dysfunction, cirrhosis, and hepatocellular carcinoma (HCC) [1, 2]. Each year, nearly 1.5 million new HBV infections occur worldwide [3], with an estimated one million HBV-related deaths, predominantly due to cirrhosis and HCC [4, 5]. The burden of HBV is disproportionately high in regions such as Sub-Saharan Africa and East Asia, where about 5 to 10% of the adult population is chronically infected [6]. Ghana exemplifies this high endemicity, with a recent systematic review reporting on HBV prevalence of 8.36% among the general adult population [1]. Among individuals with chronic liver complications, such as cirrhosis, the prevalence rises dramatically to 53.8%, as reported by Agbozo et al. (2022), underscoring the disease’s progression and its clinical impact. Despite the significant disease burden, gaps remain in understanding coinfections and occult infections that may further exacerbate liver injury in this population [7].

Occult hepatitis B infection (OBI) is defined by the presence of HBV DNA in the blood or liver of hepatitis B surface antigen (HBsAg) negative individuals [8]. Although clinically silent, OBI is linked to an increased risk of cirrhosis and HCC [9, 10].

HDV infection is also associated with an even more aggressive disease course, characterised by rapid fibrosis progression, higher risk of hepatic decompensation, and increased hepatocellular carcinoma incidence. Recent reports also highlight the clinical challenges HDV poses in pregnancy, including more severe maternal disease and higher rates of adverse outcomes [11].

Despite the clinical significance of both occult hepatitis B infection (OBI) and HBV/HDV coinfection [16, 17], there is a lack of epidemiological data on their prevalence and hepatic impact in Ghana, particularly in regions outside Greater Accra and Ashanti [12, 13]. While previous studies in Ghana have explored host genetic susceptibility [14] and non-invasive markers of liver fibrosis in HBV-infected individuals [15], few studies have investigated the burden of OBI and HDV in more rural or underserved areas. The World Health Organisation’s goal of eliminating hepatitis B by 2030 is constrained by diagnostic capacity gaps in many low-resource settings. Standard HBsAg testing may miss cases in the window period, immune-control phases, and occult HBV infection. More sensitive marker panels and pre-testing strategies are needed. Although nucleic acid testing improves detection, its cost and equipment requirements limit its widespread use in low-income regions [16].

This study aimed to determine the prevalence of HDV coinfection and OBI and to evaluate their associations with liver, inflammatory, and fibrotic biomarkers among patients attending Bimbilla Hospital, Northern Ghana.

## Materials and methods

### Study design & patients

This comparative cross-sectional study was conducted at Bimbilla Hospital in Northern Ghana between May and December 2024. The hospital, located in the Nanumba North Municipality, serves as the primary referral centre, with a catchment population of approximately 188,680. The hospital provides secondary-level healthcare services to predominantly rural farming communities and receives referrals from surrounding primary health facilities. As one of the few facilities in the area with basic laboratory capacity for viral hepatitis screening, it represents a typical rural healthcare setting in Northern Ghana where HBV burden is high, but specialised diagnostic resources are limited.

We enrolled 120 participants: 60 HBsAg-positive individuals (HBsAg-positive Group) and 60 HBsAg-negative individuals (HBsAg-negative Group). Sample size for OBI detection was calculated using the single population proportion formula [17]: S = Z²p(1-p)/d². Using Z = 1.96 for a 95% confidence level, *p* = 0.073 [18] and d = 0.05, the minimum required sample size was 104.

In the study, OBI was defined as HBsAg-negative with detectable HBV DNA, consistent with the seronegative form of OBI described in some cohorts [19]. HBV DNA quantification was not performed. Participants included patients seeking medical care at Bimbilla Hospital for various reasons, including routine check-ups and hepatitis screening. Exclusion criteria were prior HBV vaccination, hepatitis C virus (HCV) infection, and pregnancy (due to physiological changes affecting liver function tests). All participants underwent a clinical review. Any individual with a recent history (within 6 months) of acute hepatitis symptoms (e.g., jaundice, malaise, abdominal pain) was excluded.

Prior HBV vaccination status was based on participant or caregiver report, as written records were not available. HCV testing was performed for all participants, and those who tested positive were excluded. Participants were recruited consecutively until sample size targets were reached.

A structured questionnaire was developed specifically for this study to collect sociodemographic data. An English version of the questionnaire has been provided as a supplementary file.

We grouped participants according to their HBV and HDV infection status as follows:

#### Group A

HBsAg-positive and HDV-RNA-positive, irrespective of HBV DNA status.

#### Group B

HBsAg-positive and HDV RNA-negative.

#### Group C (OBI)

HBsAg-negative with detectable HBV DNA and HDV negative.

#### Group D (controls)

HBsAg-negative, HBV DNA-negative, and HDV-negative.

A control group consisting of HBsAg-negative, HBV DNA-negative, and HDV-negative participants was included to provide baseline liver biomarker values and to enable comparison with HBV- and HDV-affected groups. The HBsAg-negative population also served as the source from which OBI cases were identified.

### Data and blood sample collection

Sociodemographic and clinical data were collected via structured questionnaires administered to consenting participants. Venous blood (5 mL) was drawn from each participant into serum separator tubes (3 mL) and EDTA tubes (2 mL). Serum samples were obtained by centrifugation at 2500 g for 7 min and stored at − 20 °C until analysis.

EDTA samples were processed within 12 h of collection for haematological markers.

### Laboratory analysis

The HBV serological markers HBsAg, HBeAg, anti-HBs, total anti-HBc, and anti-HBe were screened using the HIGHTOP^®^ HBV 5-in-1 test panel (REF: H122; Qingdao Hightop Biotech, China, 2023), following the manufacturer’s instructions. Quality controls consisted of ELISA-confirmed HBV antigen and antibody samples obtained from the Noguchi Memorial Institute for Medical Research (NMIMR) at the University of Ghana.

Serum was used for HBV serological marker testing and liver function analyses, while plasma was used for nucleic acid extraction and HDV RNA detection, following the manufacturer’s recommendations. All samples were stored at − 20 °C (each batch for a maximum of 3 weeks) in a continuous cold-chain, consistent with the kit instructions and literature indicating that HDV RNA is stable at this temperature for several months when properly handled [20].

HBV DNA and HDV RNA were extracted from 400 µL of plasma using the Robogene INSTANT virus RNA/DNA isolation kit (REF: 8470259200603; Roboscreen GmbH, Germany, 2023) according to the manufacturer’s instructions. Briefly, plasma samples were lysed with proteinase K and lysis buffer, followed by binding of viral nucleic acids to a silica membrane, washing with high- and low-salt buffers, and elution in 60 µL of RNase-free water preheated to 70 °C. A synthetic internal control (IC) was added during the lysis step to monitor nucleic acid extraction efficiency, PCR amplification performance, and inhibition.

Quantitative real-time PCR detection was performed using the CFX Opus 96 Real-Time PCR System (Bio-Rad Laboratories, Canada, 2021). HBV DNA was detected using the RoboGene HBV DNA Quantification Kit 3.0 (REF: 847-0207710096), which targets the S-gene to detect all 9 genotypes (A-I), with a limit of detection (LOD) of 10.4 IU/mL. HDV RNA was detected using the RoboGene HDV RNA Quantification Kit 2.0 (REF: 847-0207400582) targeting the hepatitis delta antigen (HDAg) gene to detect all eight major genotypes [1–8], with an LOD of 14 IU/mL.

Both assays use TaqMan^®^ probe-based chemistry and fluorescence detection in the FAM channel and were performed in a 25 µL reaction volume. Each run included: [1] positive controls - WHO/PEI reference standards plus 4-level quantification standards [2], negative controls - virus-free plasma/serum, and [3] internal controls - synthetic IC to monitor extraction-to-PCR workflow as per manufacturer protocols. Both demonstrate high analytical and diagnostic specificity, as reported by the manufacturer.

Liver function tests were conducted on the URIT 810 Chemistry Analyser (URIT Medical Electronic Co., Ltd, China, 2018). Haematological parameters were measured using the Rayto RT-7600 S Auto Haematology Analyser (Rayto Life and Analytical Sciences Co., Ltd, China, 2019).

Fibrosis and inflammation indices were calculated, including: Albumin-to-Globulin Ratio (A/G), Alkaline Phosphatase to Platelet Ratio (APPR), *De Ritis ratio: AST / ALT*, Gamma-Glutamyl Transferase to Platelet Ratio (GPR): *GPR = (GGT (U/L) / ULN GGT) / Platelet count (10⁹/L)*,* ULN for GGT = 55 U/L*, and Aspartate Aminotransferase-to-Platelet Ratio Index (APRI): *APRI = ((AST / ULN) / Platelet count (10⁹/L)) × 100. ULN for AST = 40 U/L.* Others were Fibrosis-4 Index (FIB-4): *FIB-4 = (Age (years) × AST (U/L)) / (Platelet count (10⁹/L) × √ALT (U/L))*, Granulocyte-to-Lymphocyte Ratio (GLR): *GLR = Granulocyte count / Lymphocyte count*, Platelet-to-Lymphocyte Ratio (PLR): *PLR = Platelet count / Lymphocyte count*, Red Cell Distribution Width to Platelet Ratio (RPR): *RPR = RDW (%) / Platelet count (10⁹/L)* and Platelet to White Blood Cell Ratio (PWR). *PWR = Platelet count / White blood cell count*.

This study was conducted and reported in accordance with the Strengthening the Reporting of Observational Studies in Epidemiology (STROBE) guidelines for cross-sectional studies.

### Data and statistical analysis

Data were analysed using SPSS version 26 and GraphPad Prism version 8. Continuous variables were summarised as means ± standard deviations or medians (interquartile range) depending on distribution, and categorical variables as frequencies and percentages. Data normality was assessed using the Shapiro-Wilk test. Between-group comparisons for continuous variables used independent-samples t-tests for normally distributed data or Mann-Whitney U tests for non-normally distributed data, while chi-square tests were used for categorical variables. The primary analysis compared APRI and other key liver fibrosis markers between HDV RNA–positive and HDV RNA–negative participants within the HBsAg-positive group. Other subgroup comparisons were treated as secondary and interpreted as exploratory.

No data transformation was performed. We used nonparametric tests for non-normally distributed data to maintain data integrity and avoid violations of assumptions inherent to transformed-data analyses. A p-value < 0.05 was considered statistically significant. Results are presented in tables and figures. Complete case analysis was employed. We excluded participants with missing data for specific biomarkers (< 5% of the total for any single variable) from analyses involving those variables but retained them for other comparisons where data were complete. No imputation methods were used, given the small percentage of missing data.

## Results

In this study, 120 participants were included: 60 HBsAg-positive and 60 HBsAg-negative. The study included 56 females (46.7%) and 64 males (53.3%), with no significant gender differences (*p* = 0.292). The median age was 33 years (IQR 29–41) among HBsAg-positive participants and 30 years (IQR 25–38) among HBsAg-negative participants (*p* = 0.109).

Some patients with HBV infection had a family history of HBV infection, compared with those without HBV infection (19 vs. 8, *p* = 0.017) (Table 1).

**Table 1:** Sociodemographic characteristics of the study participants (*n* = 120)

Variable	HBsAg-positive (*n* = 60)	HBsAg-negative (*n* = 60)	*P*-value
**Gender**			0.292
Female	30 (50.0%)	26 (43.3%)	
Male	30 (50.0%)	34 (56.7%)	
**Age (years)**			**0.109**
Median (IQR)	**33 (29–41)**	**30 (25–38)**	
**Age groups**			
19–29	16 (26.7%)	26 (43.3%)	
30–39	26 (43.3%)	22 (36.7%)	
40–49	12 (20.0%)	5 (8.3%)	
50–59	6 (10.0%)	4 (6.7%)	
60–69	0 (0.0%)	2 (3.3%)	
70–79	0 (0.0%)	1 (1.7%)	
**Family history of HBV infection**			**0.017**
No	41 (68.3%)	52 (86.7%)	
Yes	19 (31.7%)	8 (13.3%)	

Baseline characteristics of participants by HBsAg status. HBsAg+ indicates HBV infection based on surface antigen positivity; HBsAg– indicates no detectable HBV infection on screening. *P* < 0.05 was considered statistically significant. Footnote: HBV, hepatitis B virus; HBsAg, hepatitis B surface antigen

**Fig. 1:**
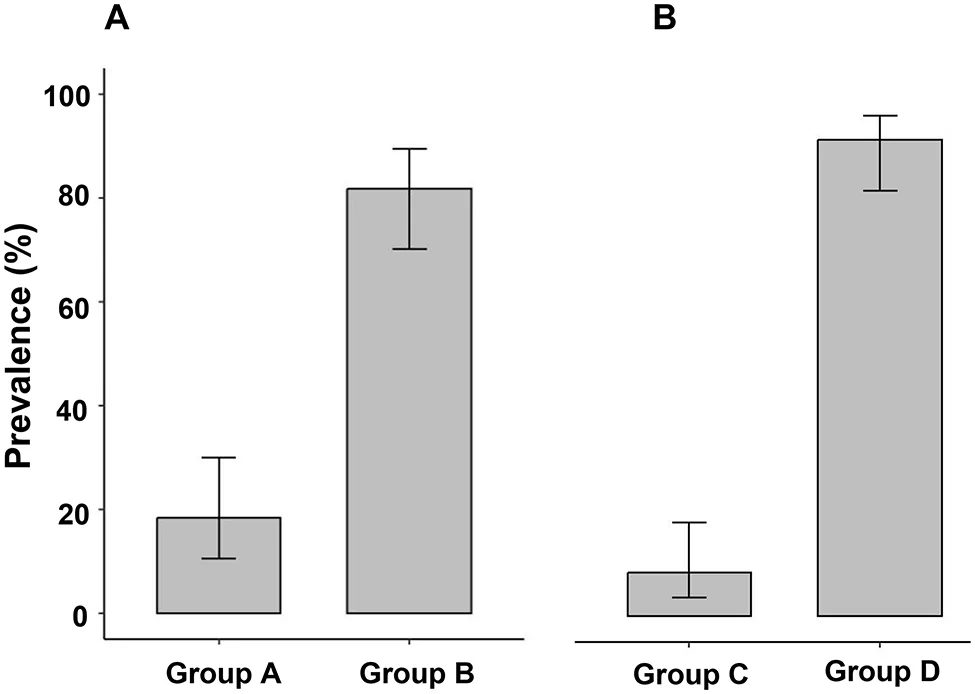
Prevalence of HDV infection among HBsAg-positive participants and occult hepatitis B infection (OBI) among HBsAg-negative participants. Panel **A** shows the prevalence of HDV RNA positivity among HBsAg-positive participants (11/60; 18.3%, 95% CI 10.6–29.9) and HDV RNA negativity (49/60; 81.7%, 95% CI 70.1–89.4). Panel **B** shows the prevalence of occult HBV infection among HBsAg-negative participants (5/60; 8.3%, 95% CI 3.6–18.1) and HBV DNA negativity (55/60; 91.7%, 95% CI 81.9–96.4). Error bars represent 95% confidence intervals calculated using the Wilson method

HDV infection was not detected in all HBsAg-negative individuals. Eleven [11] (18.3%) of the 60 HBsAg-positive participants had HDV infection, while 5 (8.3%) of the 60 HBsAg-negative participants had OBI detected by HBV DNA positivity (Fig. 1).

### Liver dysfunction markers of the study participants

Liver enzyme activities differed significantly between HBsAg-positive and HBsAg-negative Groups (Table 2). AST, GGT, and total bilirubin were elevated in HBsAg-positive patients (*p* < 0.001 for each), while ALP was inconsistently higher in HBsAg-negative controls (*p* < 0.001).

**Table 2:** Comparison of liver biomarkers and indices between patients with HBsAg-positive and those with HBsAg-negative

Biomarker or Index	HBsAg-positive *(n = 60)* Median (IQR)	HBsAg-negative *(n = 60)* Median (IQR)	*P*-value
TP (g/L)	66.87 (61.00–70.00)	67.37 (63.10-72.98)	0.129
ALB (g/L)	37.00 (35.00–39.00)	37.80 (33.33–41.40)	0.275
GLB (g/L)	29.00 (24.61-32.00)	29.03 (26.02–34.73)	0.141
AST (U/L)	43.84 (25.00-74.75)	28.16 (20.00-34.33)	**< 0.001**
ALT (U/L)	30.61 (18.58-52.00)	29.70 (25.34–37.67)	0.386
ALP (U/L)	161.67 (139.16-223.11)	108.00 (84.25–137.50)	**< 0.001**
GGT (U/L)	38.00 (36.00-47.25)	20.93 (17.50–29.00)	**< 0.001**
TBIL (µmol/L)	11.76 (9.53–16.62)	8.00 (5.12-10.00)	**< 0.001**
DBIL (µmol/L)	4.92 (3.33–8.48)	2.00 (1.00-3.98)	**< 0.001**
IBIL (µmol/L)	6.60 (4.35–8.05)	5.00 (4.00-6.79)	0.**029**
A/G Ratio	1.31 (1.16–1.47)	1.25 (1.03–1.49)	0.289
GPR	0.37 (0.27–0.56)	0.22 (0.16–0.35)	**< 0.001**
AFP (ng/mL)	3.35 (1.43–6.23)	3.10 (1.20–4.90)	0.143
De Ritis ratio	1.49 (1.09–1.88)	0.99 (0.76–1.16)	**< 0.001**
APRI	0.57 (0.37–1.07)	0.34 (0.28–0.51)	**< 0.001**
FIB-4	1.29 (0.74–2.14)	0.74 (0.55–1.14)	**< 0.001**
GLR	2.04 (1.21–2.89)	1.17 (0.66–2.47)	**0.004**
PLR	97.13 (64.54-129.45)	80.00 (58.28-119.47)	0.381
RPR	0.60 (0.50–0.82)	0.07 (0.06–0.10)	**0.018**
PWR	25.83 (17.18–40.39)	31.10 (20.69–44.60)	0.152

Liver biomarkers among HBsAg-positive cases and HBsAg-negative controls. Values are medians (interquartile ranges). A/G = albumin-to-globulin ratio, GGT = gamma-glutamyl transferase, APRI = AST-to-platelet ratio index, FIB-4 = fibrosis-4 index, GPR = GGT-to-platelet ratio, GLR = granulocyte-to-lymphocyte ratio, PLR = platelet-to-lymphocyte ratio. P < 0.05 is considered statistically significant

Fibrotic and inflammatory indices, including APRI, FIB-4, GLR, and de Ritis ratio, were significantly elevated in HBsAg-positive patients (all *p* < 0.001), indicating increased liver injury and inflammation.

### Comparison of biomarkers in HBV/HDV coinfection versus HBV mono-infection

Among HBsAg-positive patients, those with HDV coinfection (Group A, *n* = 11) showed significantly higher inflammatory markers than those without HDV (Group B, *n* = 49), as shown in Table 3.

**Table 3:** Comparison of liver biochemical, fibrotic, and inflammatory indices between HBV/HDV coinfected patients and HBV monoinfected patients

Biomarker or Index	HBV/HDV Coinfected Patients *Group A (n = 11)* Median (IQR)	HBV Monoinfected Patients *Group B (n = 49)* Median (IQR)	*P*-value
TP (g/L)	64.00 (60.00–72.00)	67.28 (61.12-70.00)	0.916
ALB (g/L)	36.00 (32.00–39.00)	37.00 (36.00–39.00)	0.179
GLB (g/L)	30.00 (27.00–33.00)	29.00 (24.17-31.00)	0.293
AST (U/L)	55.00 (44.00-101.00)	40.00 (23.23–62.90)	0.071
ALT (U/L)	47.00 (31.00–79.00)	30.00 (16.50-48.87)	0.065
ALP (U/L)	120.00 (111.00-130.00)	100.00 (81.00-141.45)	0.126
GGT (U/L)	45.00 (39.00–57.00)	37.00 (36.00-42.52)	**0.020**
TBIL (µmol/L)	13.31 (10.50-17.75)	11.30 (9.40-14.88)	0.374
DBIL (µmol/L)	6.18 (3.00-7.57)	4.40 (3.30–8.60)	0.985
IBIL (µmol/L)	7.23 (5.09–11.57)	6.60 (4.30–7.60)	0.175
A/G Ratio	1.21 (1.05–1.29)	1.34 (1.21–1.50)	**0.014**
GPR	0.42 (0.35–0.79)	0.36 (0.26–0.55)	0.248
AFP (ng/mL)	3.30 (1.40–5.35)	4.30 (3.00–8.00)	0.191
De Ritis ratio	1.76 (1.11–1.98)	1.47 (1.06–1.85)	0.330
APRI	0.63 (0.49–1.40)	0.53 (0.32–1.02)	0.129
FIB-4	1.66 (0.77–2.29)	1.15 (0.73–2.11)	0.232
GLR	2.60 (2.02–4.93)	1.87 (1.09–2.55)	**0.035**
PLR	143.00 (67.00-173.78)	89.87 (62.25-111.71)	**0.035**
RPR	0.06 (0.05–0.07)	0.07 (0.05–0.08)	0.385
PWR	45.33 (19.80-56.45)	25.80 (16.31–31.47)	0.160

Liver biomarkers among HBsAg-positive patients with HDV RNA detected (Group A) and HBsAg-positive patients without HDV RNA (Group B). Values are medians (interquartile ranges). A/G = albumin-to-globulin ratio, GGT = gamma-glutamyl transferase, APRI = AST-to-platelet ratio index, FIB-4 = fibrosis-4 index, GPR = GGT-to-platelet ratio, GLR = granulocyte-to-lymphocyte ratio, PLR = platelet-to-lymphocyte ratio. P < 0.05 is considered statistically significant

PLR was elevated in Group A (median difference: 53.1, 95% CI: 12.4–98.7, *p* = 0.035), as was GLR (median difference: 0.73, 95% CI: 0.15–1.88, *p* = 0.035). GGT activity was higher in HDV coinfection (median difference: 8.0 U/L, 95% CI: 1.5–15.2, *p* = 0.020).

### Comparison of liver function and fibrosis markers in occult hepatitis B infection and seronegative controls

This study also compared biochemical, haematological, and fibrotic indices between patients with occult hepatitis B infection (OBI), defined as HBsAg-negative, undetectable HDV RNA, but detectable HBV DNA (Group C), and those negative for HBsAg, HBV DNA, and HDV RNA (Group D).

As shown in Table 4, we did not observe any statistically significant differences between the two Groups across all measured parameters, including liver enzymes (AST, ALT, ALP, GGT), bilirubin fractions, protein levels, and fibrosis scores (APRI, FIB-4). These findings indicate comparable liver function and fibrosis status in patients with OBI and those without detectable HBV or HDV markers.

**Table 4:** Comparison of liver biochemical, fibrotic, and inflammatory indices between Group C and Group D

Biomarker or Index	*Group C (n = 5)* Median (IQR)	*Group D (n = 55)* Median (IQR)	*P*-value
TP (g/L)	66.50 (64.73–74.05)	67.64 (62.66–72.99)	0.659
ALB (g/L)	35.90 (33.65–43.40)	37.85 (33.19–41.54)	0.856
GLB (g/L)	26.82 (21.33–38.75)	29.31 (26.08–34.63)	0.585
AST (U/L)	24.90 (16.06–31.76)	28.70 (20.89–34.43)	0.370
ALT (U/L)	28.90 (19.90–33.10)	29.75 (25.36–37.92)	0.422
ALP (U/L)	144.58 (81.93–245.00)	163.57 (139.79-223.42)	0.530
GGT (U/L)	21.00 (19.36-118.28)	20.93 (16.96–28.55)	0.322
TBIL (µmol/L)	6.00 (5.00-9.38)	8.00 (5.78-10.00)	0.276
DBIL (µmol/L)	1.00 (1.00–5.00)	2.00 (1.00-3.99)	0.381
IBIL (µmol/L)	4.00 (4.00-4.88)	5.96 (4.00-5.96)	0.081
A/G Ratio	1.45 (0.91–1.86)	1.23 (1.04–1.49)	**0.**602
GPR	0.25 (0.17–1.15)	0.22 (0.16–0.35)	0.530
AFP (ng/mL)	2.00 (0.85–6.70)	3.10 (1.28–4.83)	0.947
De Ritis ratio	1.02 (0.65–1.16)	0.99 (0.75–1.16)	0.947
APRI	0.29 (0.21–0.39)	0.37 (0.27–0.53)	0.224
FIB-4	0.84 (0.59–1.04)	0.74 (0.55–1.16)	0.947
GLR	1.10 (0.52–3.73)	1.17 (0.66–2.52)	0.718
PLR	78.65 (67.52-129.25)	80.58 (57.99-119.96)	0.748
RPR	0.07 (0.06–0.07)	0.07 (0.06–0.10)	0.273
PWR	24.19 (19.81–43.39)	32.04 (20.62–44.68)	0.779

Liver biomarkers among OBI patients (Group C) and HBsAg-negative/HBV DNA-negative controls (Group D). Values are medians (interquartile ranges). All other abbreviations are as defined in Tables 2 and 3. P < 0.05 is considered statistically significant

### Comparison of liver biochemical, fibrotic, and inflammatory indices between overt HBV infection without HDV coinfection (Group B) and occult HBV infection without HDV coinfection (Group C)

Table S1 compares liver biochemical, fibrotic, and inflammatory indices between patients with overt HBV infection without HDV coinfection (Group B) and those with occult HBV infection without HDV coinfection (Group C).

While most parameters, including total protein, albumin, globulin, and liver enzymes (AST, ALT, ALP, GGT), showed no statistically significant differences, total bilirubin (TBIL) and direct bilirubin (DBIL) were significantly lower in the Group C compared to the Group B (TBIL median 6.00 vs. 11.3 µmol/L, *p* = 0.018; DBIL median 1.00 vs. 4.40 µmol/L, *p* = 0.015).

The De Ritis ratio was also significantly lower in Group C (median 1.02 vs. 1.47, *p* = 0.030), suggesting relatively less hepatocellular injury. Fibrosis scores (APRI, FIB-4) and inflammatory indices such as GLR and PLR did not differ significantly between the Groups. However, APRI showed a trend toward lower values in Group C (*p* = 0.066).

These findings suggest that patients with occult HBV infection without HDV coinfection may have less cholestatic dysfunction and reduced hepatocellular damage compared to those with overt HBV infection. At the same time, fibrosis and inflammation profiles appear comparable.

### Comparison of liver biochemical, fibrotic, and inflammatory markers in HBV/HDV coinfected patients versus occult HBV infection

Finally, we compared liver biochemical, fibrotic, and inflammatory markers between HBsAg-positive patients with HDV coinfection (Group A) and those with occult HBV infection who were HDV-negative (Group C) (Table S2).

Group A showed significantly higher aminotransferase levels, with median AST (55.00 U/L vs. 24.90 U/L, *p* = 0.020) and ALT (47.00 U/L vs. 28.90 U/L, *p* = 0.047), indicating greater hepatocellular injury. Total bilirubin (13.31 µmol/L vs. 6.00 µmol/L, *p* = 0.020) and indirect bilirubin (7.23 µmol/L vs. 4.00 µmol/L, *p* = 0.009) were also higher in Group A. The De Ritis ratio was significantly elevated in Group A (1.76 vs. 1.02, *p* = 0.036). APRI was higher in Group A (0.63 vs. 0.29, *p* = 0.004), while the difference in FIB-4 did not reach significance (*p* = 0.062). No significant differences were observed in total protein, albumin, globulin, ALP, GGT, A/G ratio, GLR, or PLR. These results show that HDV coinfection may be associated with more pronounced liver injury and fibrosis than occult HBV infection alone.

## Discussion

### Epidemiology of HBV infection in Northern Ghana

This study examined the rates of HBV/HDV coinfection, occult hepatitis B infection (OBI), and liver dysfunction markers among patients at Bimbilla Hospital in Ghana. Most HBV cases occurred in individuals aged 20–39 years, consistent with trends from Nigeria and Ethiopia [21, 22]. Because the HBV-negative group in this study was intentionally selected for comparison, their HBV-negative status reflects the study design rather than differences in exposure or vaccination.

A strong link was found between HBV-positive status and family history, highlighting intra-familial transmission as a major route of spread [23]. High maternal HBV DNA levels are an important driver of intrauterine transmission, with viral loads ≥ 10⁷ copies/mL markedly increasing fetal infection risk [24]. In addition, in rural Northern Ghana, traditional practices such as ritual scarification, circumcision performed with non-sterile instruments, and communal use of sharp tools may contribute to ongoing HBV spread [25]. Vertical and horizontal transmissions remain critical pathways sustaining HBV endemicity in Ghana, consistent with WHO reports highlighting perinatal and early childhood exposures as major drivers of HBV persistence in high-endemic settings [26].

Cameroon has documented substantially higher burdens in several studies, with anti-HDV prevalences reported across different cohorts from 10.5% (hospital/clinical series) up to 46.7% in large HBsAg-positive sample sets; for example, Luma et al. (2017) [27] reported 10.5% anti-HDV in a tertiary-hospital series, Foupouapouognigni et al. (2011) [28] found 17.6% HDV-seropositive in 233 HBsAg-positive patients, and 61% detectable HDV RNA in 25 of the 41 reported 17.6%, and Butler et al. (2018) [29] reported 46.73% seroprevalence (active HDV RNA in 34.2% of HBsAg-positives) in their multicohort Cameroonian sample. These figures illustrate the substantial within-country heterogeneity in Cameroon.

In Burkina Faso and neighbouring West African settings, Coffie et al. (2017) [30] reported 14.9% (95% CI 7.4–25.7) anti-HDV among HBsAg-positive individuals in their multicountry West-African HIV cohort. Differences between studies often reflect the sampled population (general population vs. hospital or HIV clinics), geographical hotspots, and whether antibody or RNA assays were used.

Because our study used RNA-based detection, direct comparisons with antibody-based seroprevalence studies must be made cautiously: anti-HDV (total Ig) indicates exposure and may persist after HDV clearance, whereas HDV RNA reflects current/active replication and thus a different (usually lower) prevalence of active infection [29].

### HBV/HDV coinfection prevalence in Northern Ghana

This study identified a coinfection rate of 18.33% for HBV/HDV among HBsAg-positive individuals in Northern Ghana, filling a gap in regional epidemiological data. This contrasts with the 60% HDV seroprevalence reported in Mongolia using an antibody-capture microarray assay [31], a method and epidemiologic setting that differ from RNA-based detection in West Africa but, together, highlight the wide geographic variability in HDV burden.

In Ghana, Attiku et al. (2021) [32] reported a 30.8% prevalence rate of OBI and a 3.54% HDV coinfection rate among 113 enrolled HIV/HBV coinfected patients in Korle-Bu, Greater Accra Region of Ghana. Similarly, Dzudzor et al. (2024) [18] also found that among hemodialysis patients, 7.7% had overt HBV infection, while 7.3% had OBI. In a study by Asmah et al. (2014) at the Korle-Bu Teaching, 37 Military, and the Ridge Hospitals, all in Accra, HDV seroprevalence was 11.3% using the immune assay technique.

### Biochemical impact of HBV/HDV coinfection

The pathogenesis of HBV-related liver disease involves chronic inflammation and fibrogenesis, processes that are accelerated by HDV coinfection, increasing the risk of cirrhosis and hepatocellular carcinoma (HCC) [33]. The diagnosis and prognosis of chronic hepatitis B depend on an integrated assessment of serological, virological, and biochemical markers. Diagnostic classification relies on markers such as HBsAg, HBeAg, anti-HBe, and HBV DNA to define infection status, replication activity, and disease phase. Prognostic evaluation extends beyond viral markers to include liver enzymes, platelet counts, and non-invasive fibrosis indices, which provide insight into necro-inflammatory activity and fibrosis progression.

HDV infection triggers strong hepatocyte intrinsic immunity and type I and III interferon responses. This upregulates interferon-stimulated genes and enhances innate immune cell activity, thereby promoting granulocyte recruitment and platelet mobilisation during early hepatic inflammation. These changes often reduce circulating lymphocytes while increasing granulocytes, leading to higher GLR and PLR values [34, 35].

HDV infection has been associated with weak but persistent CD8 + T-cell responses and sustained cytokine signalling, processes that may contribute to lymphocyte exhaustion and redistribution from peripheral blood to the liver. In addition, platelet activation has been reported in HDV-associated liver injury and may partly contribute to elevated PLR and hepatic inflammation observed in HDV infection [36]. Higher GGT levels in HBV/HDV coinfection reflect more severe hepatocellular injury and cholestasis, which are characteristic of the aggressive disease course seen in HDV infection [37]. Additionally, reduced albumin-to-globulin ratios and increased gamma-glutamyl transferase (GGT) levels in coinfected patients further indicate more advanced liver injury [38].

As highlighted by Hudu et al., no single marker is sufficient; rather, combining viral load, biochemical indices, and surrogate fibrosis scores improves risk stratification and the prediction of long-term outcomes, such as cirrhosis and hepatocellular carcinoma [39].

In the setting of HDV coinfection, prognostic assessment is particularly important, as HDV coinfection suggests association with more rapid liver injury and fibrosis progression, underscoring the need for closer monitoring and earlier intervention.

These principles are reflected in our findings, in which combining interpretations of virological status, inflammatory indices, and fibrosis markers provided clearer differentiation between HBV mono-infection, HBV/HDV coinfection, and OBI phenotypes.

In line with this, patients with HBV/HDV coinfection in our study exhibited elevated inflammatory markers, such as PLR and GLR (*p* = 0.035), corroborating previous findings associating these indices with HDV infection and fibrosis severity [40, 41].

Our HDV coinfection prevalence of 18.3% among HBsAg-positive patients lies within the wide range reported from West and Central Africa. In Nigeria, et al. (2021) [42] found,5.6% had anti-HDV antibodies without detectable HDV RNA among HBV-infected individuals, indicating past or resolved infection, while 16.0% had detectable HDV RNA but no anti-HDV antibodies, suggesting active HDV infection during the early seronegative phase or seronegative HDV replication, while Nwokediuko & Ijeoma (2009) [43] reported 12.5% anti-HDV among patients with HBV-related liver disease (prevalence varied by clinical subgroup: 4.3% in acute/asymptomatic infections vs. 15% in chronic liver disease/PLC).

### Occult hepatitis B infection (OBI): Prevalence and clinical significance

The 8.3% occult HBV infection (OBI) prevalence observed among HBsAg-negative individuals in our cohort is compatible with published African data, though OBI prevalence varies widely by population and study methods. A continental systematic review and meta-analysis found an overall OBI prevalence of 14.8% (pooled estimate across African studies), with seronegative OBI at 7.4%, reflecting heterogeneity by study population (blood donors, HIV-positive, CLD patients, high-risk Groups).

Country-level estimates also vary: Kenyan high-risk cohorts have reported OBI prevalences in the high-teens (e.g., Jepkemei et al. (2020) [44] reported figures around 18.7% in certain high-risk Kenyan Groups), while Ethiopian studies report lower to moderate prevalences (for example, Ayana et al. (2020) [45] found 5.8% OBI among isolated-anti-HBc individuals in eastern Ethiopia; other Ethiopian series report prevalences up to the mid-teens depending on the subgroup). Other African studies have found OBI prevalence of about 8–11% among blood donors in Nigeria [46], 7.7% in Cameroon among HIV positive patients [47], 8.5% in Botswana among ART-naïve HIV patients [48]. In Ghana, Attiku et al. (2021) [32] reported a 30.8% prevalence rate of OBI among 113 enrolled HIV/HBV coinfected patients in Korle-Bu, Greater Accra Region of Ghana. Similarly, Dzudzor et al. (2024) [18] also found that among hemodialysis patients, 7.7% had overt HBV infection, while 7.3% had OBI.

Again, all our OBI cases were seronegative occult hepatitis B infection (seronegative OBI). This is a less recognised form of occult HBV infection in which individuals have detectable HBV DNA despite testing negative for HBsAg, anti-HBc, and anti-HBs. Unlike the more common seropositive OBI, in which anti-HBc and/or anti-HBs antibodies remain detectable, seronegative OBI can easily evade routine screening and therefore represents a diagnostic challenge, particularly in resource-limited settings where molecular testing is not readily available [13, 49].

The mechanisms underlying seronegative OBI are not yet fully understood but are thought to involve very low-level viral replication, suppression of viral gene expression by host immune responses, progressive waning of HBV antibodies over time, and mutations within the S gene that reduce HBsAg detectability by conventional assays [49]. In some individuals, especially those with impaired immune responses, antibody titres may decline to undetectable levels despite persistence of low-grade infection. Because of these features, diagnosis relies almost entirely on highly sensitive nucleic acid amplification techniques such as real-time PCR [50].

Although seronegative OBI is often clinically silent, it remains clinically important. Previous studies have shown that these individuals may still serve as reservoirs for HBV transmission through blood transfusion, organ transplantation, or vertical transmission.

Thus, our 8.3% OBI sits within the spectrum described for Africa and underscores OBI’s role as a hidden HBV reservoir [51, 52].

### Comparative analysis of biochemical changes in HBV/HDV coinfection, HBV monoinfection and OBI

In the current study, biochemical analyses revealed significantly higher aminotransferase levels (AST, ALT), bilirubin fractions, and fibrosis scores (APRI) in HBV/HDV-coinfected individuals compared to HBV mono-infected patients and those with OBI, reflecting more pronounced hepatocellular damage and fibrosis progression [10, 41]. These observations align with international data demonstrating increased hepatic complications and HCC prevalence in HDV coinfection [38, 53].

Interestingly, no significant elevations in liver dysfunction markers were noted among OBI patients compared to HBsAg-negative controls, consistent with studies showing low-level viral replication in occult infection [13, 38, 53, 54]. The minimal biochemical abnormalities observed are expected, though the slightly higher GGT levels, even within the normal range, may reflect subtle hepatobiliary stress.

Importantly, the absence of overt enzyme elevations should not be interpreted as clinical insignificance. OBI has been linked to progressive liver injury, reactivation during immunosuppression, and an increased long-term risk of fibrosis and HCC in several cohorts. These considerations support the need for periodic monitoring of individuals with OBI, even when routine biochemical markers appear stable [54].

In overt HBV infection without HDV, significantly higher bilirubin levels and De Ritis ratios than in OBI patients further support the roles of active viral replication and immune-mediated injury in liver pathology [53, 54]. The WHO-endorsed non-invasive fibrosis markers APRI and FIB-4 have shown promise in identifying advanced fibrosis, particularly among patients co-infected with HBV and HDV, supporting their utility in resource-limited settings where FibroScan is unavailable [38, 53] Our findings are consistent with recent studies demonstrating that APRI and FIB-4 have enhanced predictive value in HDV-related liver disease [55].

Collectively, these findings emphasise the significant impact of HDV coinfection on liver injury and fibrosis progression among HBV-infected patients in Northern Ghana. They emphasise the importance of routine HDV screening, particularly in endemic regions, and suggest that inflammatory and fibrotic biomarkers, such as PLR, GLR, APRI, and FIB-4, could serve as cost-effective tools for risk stratification and management in settings with limited access to advanced diagnostics.

Our findings have important implications for clinical practice in resource-limited settings. The WHO guidelines for chronic hepatitis B recommend using non-invasive tests, such as APRI and FIB-4, or fibrosis assessment when elastography and biopsy are unavailable [56]. However, these indices were primarily validated in HBV or HCV mono-infections, and their performance in HDV coinfection requires further study.

Our observation that APRI and FIB-4 trended higher in HDV coinfection suggests potential utility, but locally validated cut-offs are needed. More promisingly, the readily available inflammatory markers PLR and GLR showed significant differences between HBV/HDV-coinfected and mono-infected patients, suggesting they could serve as screening tools to identify high-risk individuals warranting further evaluation or treatment prioritisation. These markers require only a basic complete blood count, which is available even at district-level laboratories. Integration of these indices into Ghana’s national HBV management guidelines could facilitate risk stratification in primary healthcare settings, enabling timely referral of high-risk patients to tertiary centres with specialised hepatology services. Cost-effectiveness studies are needed to evaluate optimal screening algorithms incorporating these biomarkers.

## Limitation

This study has several important limitations. First, the study population was restricted to individuals seeking care at Bimbilla Hospital in the Northern Region of Ghana during the study period. Consequently, the findings may not be fully generalisable to broader populations within Ghana or to individuals who do not utilise healthcare services. Because the study was hospital-based, symptomatic or health-seeking individuals may have been overrepresented, potentially inflating prevalence estimates and biomarker abnormalities compared with community-based populations.

Second, the cross-sectional design limits causal inference and prevents assessment of the temporal progression of HBV, HDV, and liver dysfunction. Longitudinal studies are therefore required to characterise disease dynamics and long-term clinical outcomes better. Third, the relatively small subgroup sizes, particularly for the OBI subgroup (*n* = 5) and HDV-positive group (*n* = 11), limited statistical power and reduced the robustness of between-group comparisons. Accordingly, subgroup analyses should be interpreted as exploratory. In addition, the large number of secondary biomarker comparisons increases the risk of type I error, and these findings should therefore be considered hypothesis-generating rather than confirmatory. Because of the small subgroup sizes, multivariable regression analyses were not performed, as adjusted models under these conditions may yield unstable parameter estimates and increase the risk of overfitting.

Several diagnostic and molecular limitations should also be acknowledged. All OBI cases were anti-HBc-negative, representing an atypical seronegative OBI phenotype. Although biological explanations may include low-level viraemia, waning antibody responses, or assay-related limitations, the possibility of misclassification or window-period infection cannot be fully excluded. In addition, anti-HBc IgM testing was not performed, limiting differentiation between occult infection and late-phase acute HBV infection.

HBV DNA quantification, HBV genotyping, and HDV genotyping were also not performed. Genotype distribution, particularly genotype E, which predominates in West Africa, and HBV DNA levels can influence disease progression and biomarker patterns [57, 58].

Finally, several potentially important clinical confounders, including comorbidities, HBeAg status, anti-HBs status, verified vaccination history, and prior HBV treatment history, were unavailable. We also did not assess prior COVID-19 infection status, which may influence immune and liver-related biomarkers [59]. These factors may influence liver enzyme levels and fibrosis markers, thereby limiting the ability to adjust for residual confounding. Although samples were processed promptly after collection, RNA degradation cannot be entirely excluded and may have contributed to the underdetection of HDV RNA in some participants.

## Conclusions

This study shows a substantial burden of occult HBV infection and HBV/HDV coinfection in Northern Ghana, with prevalences of 8.3% and 18.3%, respectively. HDV coinfection may be associated with higher median levels of liver enzymes, inflammatory markers, and fibrosis indices, indicating comparable hepatocellular injury. However, OBI cases showed no significant biochemical abnormalities, and the very small sample size (*n* = 5) limits any further inferences on this finding.

Routine HDV screening in HBV-positive individuals and the use of affordable, non-invasive fibrosis markers such as APRI, FIB-4, PLR, and GLR should be prioritised in clinical guidelines, particularly in resource-limited settings.

## Supplementary Information

Below is the link to the electronic supplementary material.

Supplementary Material 1

## Data Availability

The authors declare that data supporting the findings of this study are available from the corresponding author on reasonable request. We did not perform viral genome sequencing; however, OBI and HDV infection were diagnosed using validated real-time PCR assays for HBV DNA and HDV RNA detection.

## Abbreviations

A/G Ratio: Albumin/Globulin Ratio
AFP: Alpha-Fetoprotein
ALT: Alanine Aminotransferase
APRI: AST to Platelet Ratio Index
AST: Aspartate Aminotransferase
DBIL: Direct Bilirubin
FIB-4: Fibrosis-4 Score
GGT: Gamma-Glutamyltransferase
GLR: Granulocyte-to-Lymphocyte Ratio
HBsAg: Hepatitis B Surface Antigen
HBV: Hepatitis B Virus
HCC: Hepatocellular Carcinoma
HCV: Hepatitis C Virus
HDV: Hepatitis D Virus
IBL: Indirect Bilirubin
OBI: Occult Hepatitis B Infection
PCR: Polymerase Chain Reaction
PLR: Platelet-to-Lymphocyte Ratio
RNA: Ribonucleic Acid
RT-PCR: Reverse Transcription Polymerase Chain Reaction
SPSS: Statistical Package for Social Sciences
TBIL: Total Bilirubin

## References

[1] Abesig J, Chen Y, Wang H, Mwekele F, Irene S, Wu XY. Prevalence of viral hepatitis B in Ghana between 2015 and 2019: A systematic review and meta-analysis. PLoS ONE. 2020;15(6):1–16. 10.1371/journal.pone.0234348. PubMed PMID: 32530945. PMC7292378 32530945

[2] Efua Sdogbey, Delali V, Armah W. Seroprevalence of Hepatitis B virus infection and associated factors among health care workers in Southern Ghana. IJID Reg. 2023;6(January):84–9. 10.1016/j.ijregi.2023.01.009. 36814439 PMC9939711

[3] Zhang C, Liu Y, Zhao H, Wang G. Global patterns and trends in total burden of hepatitis B from 1990 to 2019 and predictions to 2030. Clin Epidemiol. 2022;14:1519. 10.2147/CLEP.S389853. PubMed PMID: 36540899. 36540899 PMC9760077

[4] Mehta P, Grant LM, Reddivari AKR. Viral hepatitis. StatPearls. 2024 Mar. PubMed PMID: 32119436.

[5] Younossi ZM, Yu ML, Yilmaz Y, Alswat KA, Buti M, Fernandez MIC, et al. Clinical and patient-reported outcome profile of patients with hepatitis B viral infection from the Global Liver Registry™. J Viral Hepat. 2023;30(4):335–44. 10. 1111/JVH.13800 PubMed PMID: 36601668. 36601668 10.1111/jvh.13800

[6] Gnyawali B, Pusateri A, Nickerson A, Jalil S, Mumtaz K. Epidemiologic and socioeconomic factors impacting hepatitis B virus and related hepatocellular carcinoma. World J Gastroenterol. 2022;28(29):3793. 10.3748/WJG.V28. I29.3793 PubMed PMID: 36157533. 36157533 PMC9367226

[7] Agbozo WK, Dzudzor B, Nyarko ENY, Lartey-abrahams K, Mensah NA, Tachi K. Sociodemographic and medical characteristics of liver cirrhosis deaths in a Ghana- ian tertiary hospital. 2022;56(4). 10.4314/gmj.v56i4.4 PMC10416286 37575631

[8] Delghandi S, Raoufinia R, Shahtahmasbi S, Meshkat Z, Gouklani H, Gholoobi A. An overview of occult hepatitis B infection (OBI) with emphasis on HBV vaccination. Heliyon. 2024;10(17):e37097. 10.1016/J.HELIYON.2024.E37097. 39281486 PMC11402251

[9] Shi Y, Wu YH, Wu W, Zhang WJ, Yang J, Chen Z. Association between occult hepatitis B infection and the risk of hepatocellular carcinoma: a meta-analysis. Liver Int. 2012;32(2):231–40. 10.1111/J.1478-3231.2011.02481.X. PubMed PMID: 21745272. 21745272

[10] Mak LY, Wong DKH, Pollicino T, Raimondo G, Hollinger FB, Yuen MF. Occult hepatitis B infection and hepatocellular carcinoma: Epidemiology, virology, hepatocarcinogenesis and clinical significance. J Hepatol. 2020;73(4):952–64. 10.1016/j.jhep.2020.05.042. PubMed PMID: 32504662. 32504662

[11] Wu J, Wang H, Xiang Z, Jiang C, Xu Y, Zhai G, et al. Role of viral hepatitis in pregnancy and its triggering mechanism. J Transl Int Med. 2024;12(4):344–54. 10.2478/JTIM-2024-0015/PDF. 39360164 PMC11444475

[12] Li C, Thapa D, Mi Q, Gao Y, Fu X. Disparities in hepatitis B virus healthcare service access among marginalised poor populations: a mixed-method systematic review. Infect Dis Poverty. 2024;13(1):1–31. 10.1186/S40249-024-01225-0/FIGURES/4. PubMed PMID: 39123232. 39123232 PMC11312201

[13] Saitta C, Pollicino T, Raimondo G. Occult hepatitis B virus infection: an update. Viruses. 2022;14(7). 10.3390/V14071504. PubMed PMID: 35891484. PMC9318873 35891484

[14] Nyarko E, Obirikorang C, Owiredu WKBA, Adu EA, Acheampong E, Aidoo F, et al. NTCP gene polymorphisms and hepatitis B virus infection status in a Ghanaian population. Virol J. 2020;17(1):1–8. 10.1186/s12985-020-01376-0. PubMed PMID: 32620148. 32620148 PMC7333392

[15] Nyarko ENY, Obirikorang C, Owiredu WKBA, Adu EA, Acheampong E. Assessment of the performance of haematological and non-invasive fibrotic indices for the monitoring of chronic HBV infection: a pilot study in a Ghanaian population. BMC Res Notes. 2023;16(1):1–8. 10.1186/s13104-023-06581-y. PubMed PMID: 37925465. 37925465 PMC10625242

[16] Kumar M, Pahuja S, Khare P, Kumar A. Current challenges and future perspectives of diagnosis of hepatitis B virus. Diagnostics. 2023;13(3):368. 10.3390/DIAGNOSTICS13030368. PMC9914745 36766473

[17] Charan J, Biswas T. How to calculate sample size for different study designs in medical research? Indian J Psychol Med. 2013;121–6. 10.4103/0253-7176.116232. PubMed PMID: 24049221. PMC3775042 24049221

[18] Dzudzor B, Nsowah KK, Agyemang S, Vento S, Amarh V, Boima V, et al. Overt and occult hepatitis B virus infection detected among chronic kidney disease patients on haemodialysis at a Tertiary Hospital in Ghana. PLoS ONE. 2024;19(3):e0290917. 10.1371/JOURNAL.PONE.0290917. PubMed PMID: 38437229. 38437229 PMC10911607

[19] Raimondo G, Locarnini S, Pollicino T, Levrero M, Zoulim F, Lok AS, et al. Update of the statements on biology and clinical impact of occult hepatitis B virus infection. J Hepatol. 2019;71(2):397–408. 10.1016/J.JHEP.2019.03.034. PubMed PMID: 31004683. 31004683

[20] Roboscreen Diagnostics. Instructions for use RoboGene HBV DNA quantification Kit 3.0 [Internet]. 2022 Jan. Available from: https://www.roboscreen.com.

[21] Akabuike OMA, Aworh MK, Uzoebo NL, Erwat J, Agukwe O, Ngong K, et al. Evaluating hepatitis B screening, prevalence, vaccination coverage, and linkage to care in Abuja, Nigeria: insights from a cross-sectional study. BMC Public Health. 2024;24(1):1–11. 10.1186/S12889-024-21017-3/FIGURES/4. 39696217 PMC11657572

[22] Larebo YM, Anshebo AA, Behera SK, Gopalan N. Prevalence of hepatitis B virus infection and associated factors among adults intrafamilial household contacts attending antenatal care clinics in the Central Ethiopian region: from pregnant women index cases. Virol J. 2025;22(1):1–11. 10.1186/S12985-025-02633-W/TABLES/5. 39939946 PMC11823231

[23] Athalye S, Khargekar N, Shinde S, Parmar T, Chavan S, Swamidurai G, et al. Exploring risk factors and transmission dynamics of Hepatitis B infection among Indian families: Implications and perspective. J Infect Public Health. 2023;16(7):1109–14. 10.1016/J.JIPH.2023.05.003. PubMed PMID: 37224621. 37224621

[24] Yin Y, zhu, Chen X, wei, Li X, mao, Hou H, ying, Shi Z. jie. [Intrauterine HBV infection: risk factors and impact of HBV DNA]. Nan Fang Yi Ke Da Xue Xue Bao. 2006;26(10):1452–4. PubMed PMID: 17062350. 17062350

[25] Tazinkeng NN, Teuwafeu DG, Asombang AW, Agbor VN, Bloom SM, Nkhoma AN, et al. Factors associated with hepatitis B and C among adults in Buea, Cameroon: A community-based cross-sectional study. Liver Int. 2022;42(11):2396–402. 10. 1111/LIV.15390 PubMed PMID: 35946051. 35946051 10.1111/liv.15390

[26] Martinson FEA, Weigle KA, Royce RA, Weber DJ, Suchindran CM, Lemon SM. Risk Factors for Horizontal Transmission of Hepatitis B Virus in a Rural District in Ghana. Am J Epidemiol. 1998;147(5):478–87. 10.1093/. OXFORDJOURNALS.AJE.A009474 PubMed PMID: 9525535. 9525535 10.1093/oxfordjournals.aje.a009474

[27] Luma HN, Eloumou SAFB, Okalla C, Donfack-Sontsa O, Koumitana R, Malongue A, et al. Prevalence and Characteristics of Hepatitis Delta Virus Infection in a Tertiary Hospital Setting in Cameroon. J Clin Exp Hepatol. 2017;7(4):334–9. 10.1016/j.jceh.2017.05.010. 29234199 PMC5715454

[28] Foupouapouognigni Y, Noah DN, Sartre MT, Njouom R. High prevalence and predominance of hepatitis delta virus genotype 1 infection in Cameroon. J Clin Microbiol. 2011;49(3):1162–4. 10.1128/JCM.01822-10. PubMed PMID: 21209162. 21209162 PMC3067734

[29] Butler EK, Rodgers MA, Coller KE, Barnaby D, Krilich E, Olivo A, et al. High prevalence of hepatitis delta virus in Cameroon. Sci Rep. 2018;8(1). 10.1038/s41598-018-30078-5. PubMed PMID: 30072752. PMC6072717 30072752

[30] Coffie PA, Tchounga BK, Bado G, Kabran M, Minta DK, Wandeler G, et al. Prevalence of hepatitis B and delta according to HIV-type: a multicountry cross-sectional survey in West Africa. BMC Infect Dis. 2017;17(1). 10.1186/s12879-017-2568-5. PubMed PMID: 28676076. PMC5496401 28676076

[31] Chen X, Oidovsambuu O, Liu P, Grosely R, Elazar M, Winn VD, et al. A novel quantitative microarray antibody capture (Q-MAC) assay identifies an extremely high HDV prevalence amongst HBV infected Mongolians. Hepatology. 2017;66(6):1739. 10. 1002/HEP.28957 PubMed PMID: 27880976. 27880976 10.1002/hep.28957 PMC5441964

[32] Attiku K, Bonney J, Agbosu E, Bonney E, Puplampu P, Ganu V, et al. Circulation of hepatitis delta virus and occult hepatitis B virus infection amongst HIV/HBV coinfected patients in Korle-Bu, Ghana. PLoS ONE. 2021;16(1 January):1–16. 10.1371/journal.pone.0244507. PubMed PMID: 33411715. PMC7790253 33411715

[33] Garbuzenko DV. Pathophysiological mechanisms of hepatic stellate cells activation in liver fibrosis. World J Clin Cases. 2022;10(12):3662–76. 10.12998/WJCC.V10. I12.3662 PubMed PMID: 35647163. 35647163 PMC9100727

[34] Dandri M, Bertoletti A, Lütgehetmann M. Innate immunity in hepatitis B and D virus infection: consequences for viral persistence, inflammation, and T cell recognition [Internet]. 10.1007/s00281-021-00864-x/Published. PMC8443521 34019142

[35] Bockmann JH, Allweiss L, Volmari A, da Fonseca Araújo D, Kohsar M, Hyrina A, et al. Hepatitis D virus infection triggers CXCL9-11 upregulation in hepatocytes and liver infiltration of CXCR3 + CD4 T cells. JHEP Rep. 2025;7(3). 10.1016/j.jhepr.2024.101273. PMC11840482 39980752

[36] Dias J, Hengst J, Parrot T, Leeansyah E, Lunemann S, Malone DFG, et al. Chronic hepatitis delta virus infection leads to functional impairment and severe loss of MAIT cells. J Hepatol. 2019;71(2):301–12. 10.1016/j.jhep.2019.04.009. PubMed PMID: 31100314. 31100314 PMC6642010

[37] Everhart JE, Wright EC. Association of γ-glutamyl transferase (GGT) activity with treatment and clinical outcomes in chronic hepatitis C (HCV). Hepatology. 2013;57(5):1725–33. 10.1002/hep.26203. PubMed PMID: 23258530. 23258530 PMC3624035

[38] Gherlan GS. Occult hepatitis B — the result of the host immune response interaction with different genomic expressions of the virus. World J Clin Cases. 2022;10(17):5518. 10.12998/WJCC.V10.I17.5518. PubMed PMID: 35979101. 35979101 PMC9258381

[39] Hudu SA, Shinkafi SH, Jimoh AO. A critical review of diagnostic and prognostic markers of chronic hepatitis B infection. Med Rev. 2024;4(3):225–34. 10.1515/mr-2024-0022. PMC11195425 38919396

[40] Wong DKH, Cheng SCY, Mak LLY, To EWP, Lo RCL, Cheung TT, et al. Among patients with undetectable hepatitis B surface antigen and hepatocellular carcinoma, a high proportion has integration of HBV DNA into hepatocyte DNA and no cirrhosis. Clin Gastroenterol Hepatol. 2020;18(2):449–56. 10.1016/j.cgh.2019.06.029. PubMed PMID: 31252193. 31252193

[41] Rizvi M, Azam M, Sultan A, Shukla I, Malik A, Ajmal MR, et al. Prevalence of genotype D in chronic liver disease patients with occult HBV infection in northern region of India. Indian J Pathol Microbiol. 2014;57(4):537–41. 10.4103/0377-4929.142643. PubMed PMID: 25308003. 25308003

[42] Opaleye OO, Akanbi OA, Osundare FA, Wang B, Adesina O, Oluremi AS, et al. Prevalence and characteristics of hepatitis B and D virus infections among HIV-positive individuals in Southwestern Nigeria. Virol J. 2021;18(1). 10.1186/s12985-021-01493-4. PubMed PMID: 33446224. PMC7809746 33446224

[43] Nwokediuko SC. Seroprevalence of antibody to HDV in Nigerians with hepatitis B virus-related liver diseases. J Clin Pract. 2009. 20329688

[44] Jepkemei KB, Ochwoto M, Swidinsky K, Day J, Gebrebrhan H, McKinnon LR, et al. Characterisation of occult hepatitis B in highrisk populations in Kenya. PLoS ONE. 2020;15(5). 10.1371/journal.pone.0233727. PubMed PMID: 32463824. PMC7255601 32463824

[45] Ayana DA, Mulu A, Mihret A, Seyoum B, Aseffa A, Howe R. Occult hepatitis B virus infection among HIV negative and positive isolated anti-HBc individuals in eastern Ethiopia. Sci Rep. 2020;10(1). 10.1038/s41598-020-79392-x. PubMed PMID: 33335238. PMC7747707 33335238

[46] Nna E, Mbamalu C, Ekejindu I. Occult hepatitis B viral infection among blood donors in South–Eastern Nigeria. Pathog Glob Health. 2014;108(5):223–8. 10.1179/2047773214Y.0000000144. PubMed PMID: 24995918. 24995918 PMC4153823

[47] Gachara G, Magoro T, Mavhandu L, Lum E, Kimbi HK, Ndip RN, et al. Characterisation of occult hepatitis B virus infection among HIV positive patients in Cameroon. AIDS Res Ther. 2017;14(1). 10.1186/s12981-017-0136-0. PubMed PMID: 28270215. PMC5341455 28270215

[48] Anderson M, Phinius BB, Phakedi BK, Mudanga M, Bhebhe LN, Tlhabano GN, et al. Persistence and risk factors of occult hepatitis B virus infections among antiretroviral therapy-naïve people living with HIV in Botswana. Front Microbiol. 2024;15. 10.3389/fmicb.2024.1342862. PMC11112038 38784816

[49] Pollicino T, Raimondo G. Occult hepatitis B, infection “Overt” HBV infection overt HBV infection occult HBV infection occult HBV infection hepatology snapshot. J Hepatol. 2014.

[50] de Almeida NAA, de Paula VS. Occult Hepatitis B virus (HBV) infection and challenges for hepatitis elimination: a literature review. J Appl Microbiol. John Wiley and Sons Inc; 2022. pp. 1616–35. 10.1111/jam.15351. PubMed PMID: 34724308. 34724308

[51] Ondigui JLN, Kenmoe S, Kengne-Ndé C, Ebogo-Belobo JT, Takuissu GR, Kenfack-Momo R, et al. Epidemiology of occult hepatitis B and C in Africa: a systematic review and meta-analysis. J Infect Public Health. 2022;15(12):1436–45. 10.1016/j.jiph.2022.11.008. PubMed PMID: 36395668. 36395668 PMC7613883

[52] Khalid E, Abrar A, Abdel Rahim MEH, Islam ME, Khalid E. Occult hepatitis B virus infection in Sudan: a systematic review and meta-analysis. JGH Open. 2020;4(5):800–7. 10.1002/jgh3.12411 PMC7578306 33102748

[53] Fattovich G, Giustina G, Christensen E, Pantalena M, Zagni I, Realdi G, et al. Influence of hepatitis delta virus infection on morbidity and mortality in compensated cirrhosis type B. Gut. 2000;46(3):420–6. PubMed PMID: 10673308. 10673308 10.1136/gut.46.3.420 PMC1727859

[54] Coghill S, McNamara J, Woods M, Hajkowicz K. Epidemiology and clinical outcomes of hepatitis delta (D) virus infection in Queensland, Australia. Int J Infect Dis. 2018;74:123–7. 10.1016/j.ijid.2018.07.005. PubMed PMID: 30003953. 30003953

[55] Wangensteen KJ, Chang KM. Multiple roles for hepatitis B and C viruses and the host in the development of hepatocellular carcinoma. Hepatology. 2021;73(S1):27–37. 10.1002/HEP.31481. PubMed PMID: 32737895. 32737895 PMC7855312

[56] WHO. Guidelines for the prevention, diagnosis, care and treatment for people with chronic hepatitis B infection [Internet]. Geneva; 2024 Mar [cited 2024 Sep 5]. Available from: https://creativecommons.org/licenses/by-nc-sa/3.0/igo. 40424433

[57] Toyé RM, Loureiro CL, Jaspe RC, Zoulim F, Pujol FH, Chemin I. The hepatitis B virus genotypes E to J: the overlooked genotypes. Microorganisms. Multidisciplinary Digital Publishing Institute (MDPI); 2023. 10.3390/microorganisms11081908. PMC10459053 37630468

[58] Cuenca-Gómez JÁ, Lozano-Serrano AB, Cabezas-Fernández MT, Soriano-Pérez MJ, Vázquez-Villegas J, Estévez-Escobar M, et al. Chronic hepatitis B genotype e in African migrants: response to nucleos(t)ide treatment in real clinical practice. BMC Infect Dis. 2018;18(1). 10.1186/s12879-018-3469-y. PubMed PMID: 30428845. PMC6236963 30428845

[59] Hyug Choi J, Sook Jun M, Yong Jeon J, Kim H, Kyung Kim Y, Ho Jeon C, et al. Global lineage evolution pattern of sars-cov-2 in Africa, America, Europe, and Asia: A comparative analysis of variant clusters and their relevance across continents. J Transl Intern Med. 2023;11(4):350–62. 10.2478/JTIM-2023-0118. 38130632 PMC10732492

