# Supplementary material for: Prevalence and biomarker profiles of HBV/HDV coinfection and occult HBV infection among patients attending Bimbilla Hospital, Northern Ghana
Source: BMC Infect Dis. 2026 Jun 25;26:1635. doi: 10.1186/s12879-026-13881-8 (PMC13563748; doi:10.1186/s12879-026-13881-8)
Supplement: Supplementary file 1 [file 12879_2026_13881_MOESM1_ESM.docx]

**Table S1.** Levels of Liver Biochemical, Fibrotic and Inflammatory Indices between Patients with Overt HBV Infection without HDV Infection (Group B) and those with Occult HBV Infection but without HDV Infection (Group C)

| ***Biomarker or***  ***Index*** | ***Group B (n=49)***  ***Median (IQR)*** | ***Group C (n=5)***  ***Median (IQR)*** | ***P-value*** |
| --- | --- | --- | --- |
| TP (g/L) | 67.28 (61.12-70.00) | 66.50 (64.73-74.05) | 0.411 |
| ALB (g/L) | 37.00 (36.00-39.00) | 35.90 (33.65-43.40) | 0.559 |
| GLB (g/L) | 29.00 (24.17-31.00) | 26.82 (21.33-38.75) | 0.917 |
| AST (U/L) | 40.00 (23.23-62.90) | 24.90 (16.06-31.76) | 0.086 |
| ALT (U/L) | 30.00 (16.50-48.87) | 28.90 (19.90-33.10) | 0.846 |
| ALP (U/L) | 100.00 (81.00-141.45) | 144.58 (81.93-245.00) | 0.194 |
| GGT (U/L) | 37.00 (36.00-42.52) | 21.00 (19.36-118.28) | 0.164 |
| TBIL (μmol/L) | 11.30 (9.4-14.88) | 6.00 (5.00-9.38) | **0.018** |
| DBIL (μmol/L) | 4.40 (3.30-8.60) | 1.00 (1.00-5.00) | **0.015** |
| IBIL (μmol/L) | 6.60 (4.30-7.60) | 4.00 (4.00-4.88) | 0.064 |
| A/G Ratio | 1.34 (1.21-1.50) | 1.45 (0.91-1.86) | 0.964 |
| AGPR | 0.77 (0.53-1.08) | 1.09 (0.88-1.18) | 0.117 |
| GPR | 0.36 (0.26-0.55) | 0.25 (0.17-1.15) | 0.165 |
| AFP (ng/mL) | 3.30 (1.40-5.35) | 2.00 (0.85-6.70) | 0.698 |
| De Ritis ratio | 1.47 (1.06-1.85) | 1.02 (0.65-1.16) | **0.030** |
| APRI | 0.53 (0.32-1.03) | 0.29 (0.21-0.39) | 0.066 |
| FIB-4 | 1.15 (0.73-2.11) | 0.84 (0.59-1.04) | 0.110 |
| GLR | 1.87 (1.09-2.55) | 1.10 (0.52-3.73) | 0.216 |
| PLR | 89.87 (62.25-111.71) | 78.65 (67.52-129.25) | 0.964 |
| RPR | 0.07 (0.05-0.08) | 0.07 (0.06-0.07) | 0.870 |
| PWR | 25.80 (16.31-31.47) | 24.19 (19.81-43.39) | 0.665 |

***Table S1****:* Liver biomarkers, fibrosis scores, and inflammatory indices among HBsAg-positive/HDV-negative patients (Group B) and OBI patients (Group C). Values are medians (interquartile ranges). APRI was calculated using the formula: (AST / AST upper limit of normal) × 100 / platelet count (10⁹/L). The AST ULN applied in this study was **40 U/L** All other abbreviations are as defined in Tables 2 and 3. P < 0.05 Considered Statistically Significant.

| ***Biomarker or***  ***Index*** | **Group A *(n=11)***  ***Median (IQR)*** | **Group C *(n=5)***  ***Median (IQR)*** | ***P-value*** |
| --- | --- | --- | --- |
| TP (g/L) | 64.00 (60.00-72.00) | 66.50 (64.73-74.05) | 0.336 |
| ALB (g/L) | 36.00 (32.00-39.00) | 35.90 (33.65-43.40) | 0.691 |
| GLB (g/L) | 30.00 (27.00-33.00) | 26.82 (21.33-38.75) | 0.691 |
| AST (U/L) | 55.00 (44.00-101.00) | 24.90 (16.06-31.76) | **0.020** |
| ALT (U/L) | 47.00 (31.00-79.00) | 28.90 (19.90-33.10) | **0.047** |
| ALP (U/L) | 120.00 (111.00-130.00) | 144.58 (81.93-245.00) | 0.335 |
| GGT (U/L) | 45.00 (39.00-57.00) | 21.00 (19.36-118.28) | 0.062 |
| TBIL (μmol/L) | 13.31 (10.50-17.75) | 6.00 (5.00-9.38) | **0.020** |
| DBIL (μmol/L) | 6.18 (3.00-7.57) | 1.00 (1.00-5.00) | **0.**068 |
| IBIL (μmol/L) | 7.23 (5.09-11.57) | 4.00 (4.00-4.88) | 0.**009** |
| A/G Ratio | 1.21 (1.05-1.29) | 1.45 (0.91-1.86) | 0.396 |
| AGPR | 0.82 (0.75-1.00) | 1.09 (0.88-1.18) | 0.234 |
| GPR | 0.42 (0.35-0.79) | 0.25 (0.17-1.15) | 0.100 |
| AFP (ng/mL) | 3.30 (1.40-5.35) | 2.00 (0.85-6.70) | 0.234 |
| De Ritis ratio | 1.76 (1.11-1.98) | 1.02 (0.65-1.16) | **0.036** |
| APRI | 0.63 (0.49-1.40) | 0.29 (0.21-0.39) | 0.**004** |
| FIB-4 | 1.66 (0.77-2.29) | 0.84 (0.59-1.04) | 0.062 |
| GLR | 2.60 (2.02-4.93) | 1.10 (0.52-3.73) | 0.079 |
| PLR | 143.00 (67.00-173.78) | 78.65 (67.52-129.25) | 0.234 |
| RPR | 0.06 (0.05-0.07) | 0.07 (0.06-0.07) | 0.157 |
| PWR | 45.33 (19.80-56.45) | 24.19 (19.81-43.39) | 0.396 |

**Table S2.** Levels of Liver Biochemical, Fibrotic and Inflammatory Indices between Patients with HBV Infected Patients Coinfected with HDV and those with Occult HBV Infection but without HDV Infection

Table S2**.** Liver biomarkers, fibrosis scores, and inflammatory indices among HBsAg-positive/HDV-positive patients (Group A) and OBI patients (Group C). Values are medians (interquartile ranges). APRI was calculated using the formula: (AST / AST upper limit of normal) × 100 / platelet count (10⁹/L). The AST ULN applied in this study was **40 U/L**. All other abbreviations are as defined in Tables 2 and 3. P < 0.05 Considered Statistically Significant.
